# Accuracy of reference air kerma in estimating incident air kerma at skin in interventional neuroradiology: Effect of X‐ray field size and alternative PERP corrections

**DOI:** 10.1002/acm2.70903

**Published:** 2026-11-11

**Authors:** Atsushi Fukuda, Nao Ichikawa, Takuma Hayashi, Pei‐Jan Paul Lin, Ayaka Hirosawa, Kosuke Matsubara

**Affiliations:** ^1^ Department of Radiological Sciences, School of Health Sciences Fukushima Medical University Fukushima Fukushima Japan; ^2^ Department of Radiological Technology, Faculty of Health Science Kobe Tokiwa University Kobe Hyogo Japan; ^3^ Department of Radiology Shiga Psychiatric Medical Center Kusatsu Shiga Japan; ^4^ Department of Radiology Virginia Commonwealth University Health System Richmond Virginia USA; ^5^ Department of Medical Technology Toyama Prefectural Central Hospital Toyama Toyama Japan; ^6^ Department of Quantum Medical Technology, Faculty of Health Sciences, Institute of Medical, Pharmaceutical and Health Sciences Kanazawa University Kanazawa Ishikawa Japan

**Keywords:** incident air kerma at skin, interventional neuroradiology, kerma‐area product, reference air kerma

## Abstract

**Background:**

Kerma‐area product (KAP) meters monitor reference air kerma ($K_{a,r}$) and KAP ($P_{KA})$. While $K_{a,r}^{KAP,Hybrid}$ is calculated using $P_{KA}$ and collimator positions, $K_{a,r}^{KAP,Meas.}$ is directly measured via a small central ionization chamber. Although $K_{a,r}$ is a common surrogate for incident air kerma at the skin ($K_{a,skin}$), its clinical application to interventional neuroradiological procedures carries higher uncertainty than regular interventional radiology, with source‐to‐skin distance being the greatest factor due to the smaller head size. Additionally, $K_{a,r}$ may not perfectly represent actual $K_{a,skin}$ due to variations in beam quality, filtration, and X‐ray field size.

**Purpose:**

This study investigated (1) the accuracy of $K_{a,r}^{KAP,Hybrid}$ and $K_{a,r}^{KAP,Meas.}$, and (2) the feasibility of employing them as surrogates for $K_{a,skin}$ during cerebral angiography and interventional neuroradiological procedures.

**Methods:**

A solid‐state dosimeter (AGMS‐DM+) was placed at the patient entrance reference point (PERP) on a polymethyl methacrylate phantom to measure $K_{a,r}^{AGMS-DM+}$. Accuracies of $K_{a,r}^{KAP,Hybrid}$ and $K_{a,r}^{KAP,Meas.}$ were evaluated against $K_{a,r}^{AGMS-DM+}$ in two investigations. First, effects of tube voltage and spectral shaping filters (1.2 mmAl, 0.03 mmTa, and 0.2 mmCu) were evaluated with/without equalization filters inserted symmetrically by 2.5 cm from both sides at a fixed X‐ray field size of 10 × 10 cm^2^. Second, the effect of varying the X‐ray field size was investigated for each filter. Additionally, a fluoroscopic system and a head phantom were modeled using computer‐aided design software. Three fields of view (FOVs) were evaluated to simulate different clinical stages: 30 × 30 cm^2^ (for whole angiography), 15 × 15 cm^2^, and 3.8 × 3.8 cm^2^ (for targeted interventional neuroradiology). Target centers were aligned with the brain center and precallosal segment of the anterior cerebral artery. Six typical clinical projections were used per FOV to determine source‐to‐skin distance and X‐ray field size at the PERP, allowing calculation of the skin‐level errors for both $K_{a,r}^{KAP,Hybrid}$ and $K_{a,r}^{KAP,Meas.}$. The effects of the corrections were also investigated using the X‐ray field size measured by $P_{KA}$/$K_{a,r}^{KAP,Meas.}$ and an alternative PERP (aPERP) positioned 11 cm from the isocenter toward the X‐ray focus.

**Results:**

Relative errors for both methods as functions of tube voltage across the three filters were within ± 4.0%. With equalization filters, $K_{a,r}^{KAP,Hybrid}$ errors ranged from −49.0%–−33.9%, exceeding the acceptable accuracy tolerance (± 35%), whereas $K_{a,r}^{KAP,Meas.}$ errors (−15.8%–−10.8%) remained within the tolerance. The relative errors of $K_{a,r}^{KAP,Hybrid}$ and $K_{a,r}^{KAP,Meas.}$ as a function of the X‐ray field size ranged from −9.7%–12.7% and −58.3%–26.1%, respectively. Although most skin‐level errors for both $K_{a,r}^{KAP,Hybrid}$ and $K_{a,r}^{KAP,Meas.}$ remained within the ± 35% tolerance in clinical simulation, those for $K_{a,r}^{KAP,Meas.}$ at the 3.8 × 3.8 cm^2^ FOV exceeded it. However, with the X‐ray field size and aPERP corrections, the errors for $K_{a,r}^{KAP,Meas.}$ were reduced to within ± 2.7%.

**Conclusions:**

$K_{a,r}^{KAP,Hybrid}$ and $K_{a,r}^{KAP,Meas.}$ should be strategically selected based on the specific clinical application, such as standard cerebral angiography versus targeted interventional neuroradiology. Particularly, $K_{a,r}^{KAP,Meas.}$ provides superior accuracy when equalization filters are used or when appropriate corrections for X‐ray field size and aPERP are applied in small‐field dosimetry.

## 1 INTRODUCTION

The first successful cerebral angiography was performed by António Egas Moniz and Pedro Almeida Lima in 1927.^1^, ^2^ Historical breakthroughs and innovations, such as the development of contrast media, wires, catheters, stents, and fluoroscopic systems, have made it possible to perform not only cerebral angiography but also fluoroscopy‐guided treatment procedures while significantly reducing complications.^2^ However, with the increasing number of interventional neuroradiological procedures, several cases of radiation‐induced skin damage, such as skin erythema and hair loss, have been reported.^3^, ^4^ Noticeable skin injuries are observed approximately 1 month after a peak skin dose exceeding several grays.^3^ Furthermore, skin damage can be caused by cumulative dose from multiple diagnostic or interventional procedures, each of which may be individually insufficient to cause injury.^5^ Therefore, radiation dose monitoring and recording during these procedures are essential to minimize radiation‐induced skin injuries.^4^

Angiographic systems are equipped with a physical or logical kerma‐area product (KAP) meter to monitor reference air kerma ($K_{a,r}^{KAP}$) and its rate (${\dot{K}}_{a,r}^{KAP}$) at patient entrance reference point (PERP) and KAP ($P_{KA}$) and its rate (${\dot{P}}_{KA}$) during interventional neuroradiological procedures.^6^ The PERP is defined at a distance of 15 cm from the isocenter to the X‐ray focus along the central beam axis.^6^ Conventional KAP meters consist of a single large‐area parallel‐plate ionization chamber to measure $P_{KA}$.^7^ $K_{a,r}^{KAP}$ can be calculated using $P_{KA}$ and X‐ray field size at the PERP as follows:

(1) $$K_{a,r}^{KAP}=\frac{P_{KA}}{X-ray\;field\;size}$$

where X−rayfieldsize is determined using the source–to–PERP distance (SPD) and digitally recorded X‐ray collimator positions. Additionally, there are physical KAP meters with a small ionization chamber located at the center (chamber‐in‐chamber). Given that the chamber‐in‐chamber KAP meter comprises two independent chambers, $K_{a,r}^{KAP}$ and $P_{KA}$ can be simultaneously measured during interventional neuroradiological procedures. $K_{a,r}^{KAP,Hybrid}$ ($K_{a,r}^{KAP}$ calculated from Equation (1)) and $K_{a,r}^{KAP,Meas.}$ ($K_{a,r}^{KAP}$ measured with a small chamber located at the center of the KAP meter) are frequently used to compare the radiation doses in interventional neuroradiological procedures.^8^ Additionally, $K_{a,r}^{KAP,Hybrid}$ and $K_{a,r}^{KAP,Meas.}$ are verified by comparing the independently measured air kerma at the PERP ($K_{a,r}$).^9^ However, because $K_{a,r}$ is not the incident air kerma at the skin ($K_{a,skin}$), the inverse square law must be applied to determine $K_{a,skin}$ as follows:

(2) $$K_{a,skin}=K_{a,r}\frac{SPD^{2}}{SSD^{2}}$$

where SSD is the source–to–skin distance. Because the examination table and C‐arm angulations are moved based on the target anatomy, determining the SSD is not a simple task in clinical settings.

Recently, X‐ray beam modeling based on computer‐aided design has been used for detailed visualization and calculation of X‐ray field sizes and overlap regions on the patient's skin during percutaneous coronary intervention and spiral CT scanning.^10^, ^11^ Based on this technique, X‐ray field size at the PERP and SSD can be evaluated based on C‐arm angulations and patient position during interventional neuroradiological procedures. Furthermore, adjusting the tube voltage, spectral shaping filters, equalization filters, and the X‐ray field size enables visualization of the entire cerebral vasculature for diagnostic procedures, as well as its small branches during interventional neuroradiological procedures. These parameter selections also dictate the measurement accuracy of $K_{a,r}$.^12^ When evaluating $K_{a,skin}$ in interventional neuroradiological procedures, $K_{a,r}$ measurement uncertainties can be further amplified due to the smaller size of the patient's head relative to the torso. Therefore, this study aimed to investigate (1) the accuracy of $K_{a,r}^{KAP,Hybrid}$ and $K_{a,r}^{KAP,Meas.}$ at the PERP as functions of tube voltage, spectral shaping filters, equalization filters, and X‐ray field size, thereby directly comparing their applicability. Furthermore, we evaluated (2) the feasibility of using these two metrics as surrogates for $K_{a,skin}$ to establish optimized dose‐monitoring strategies tailored to both whole‐cerebral angiography and highly collimated interventional neuroradiological procedures.

## 2 METHODS

### 2.1 Devices

A C‐arm fluoroscopic system (Ultimax‐i, Canon Medical Systems, Otawara, Japan) equipped with a chamber‐in‐chamber KAP meter (DIAMENTOR RS‐KDK, PTW Freiburg, Germany) was used to verify the accuracy of $K_{a,r}^{KAP,Hybrid}$ and $K_{a,r}^{KAP,Meas.}$. The built‐in chamber‐in‐chamber KAP meter was calibrated under the American Association of Physicists in Medicine (AAPM) TG 190 protocol before data collection.^9^ The SPD of the fluoroscopic system was 56 cm, and the flat‐panel detector (FPD) had a maximum field of view (FOV) of 42 × 42 cm^2^. The X‐ray tube could be positioned above or below the patient, and the system supported a tube voltage range of 50–115 kV and a tube current range of 10–100 mA. The spectral shaping filter (SSF) installed in the system comprised a 1.2‐mm‐thick aluminum (Al) filter, a 0.03‐mm‐thick tantalum (Ta) filter, and a 0.2‐mm‐thick copper (Cu) filter.

A custom‐built phantom was developed to align the X‐ray field size at the PERP. The phantom size was 40 × 40 × 2 cm^3^ and was made of polymethyl methacrylate (PMMA) (Figure 1). 2‐mm‐wide and 5‐mm‐deep grooves were milled on both sides of a 20‐mm‐thick PMMA plate (40 × 40 cm^2^). The square grooves had side lengths of 2.5, 5.0, 7.5, 10, 15, 20, 25, 30, and 35 cm. Because these square lines were milled on both the front and back surfaces, wide X‐ray fields in fluoroscopy produced double lines on the projection images. Consequently, the X‐ray field at the mid‐depth of the phantom was indicated by the center of these double lines. In this study, this custom phantom is referred to as the PMMA sizing plate.

**FIGURE 1:**
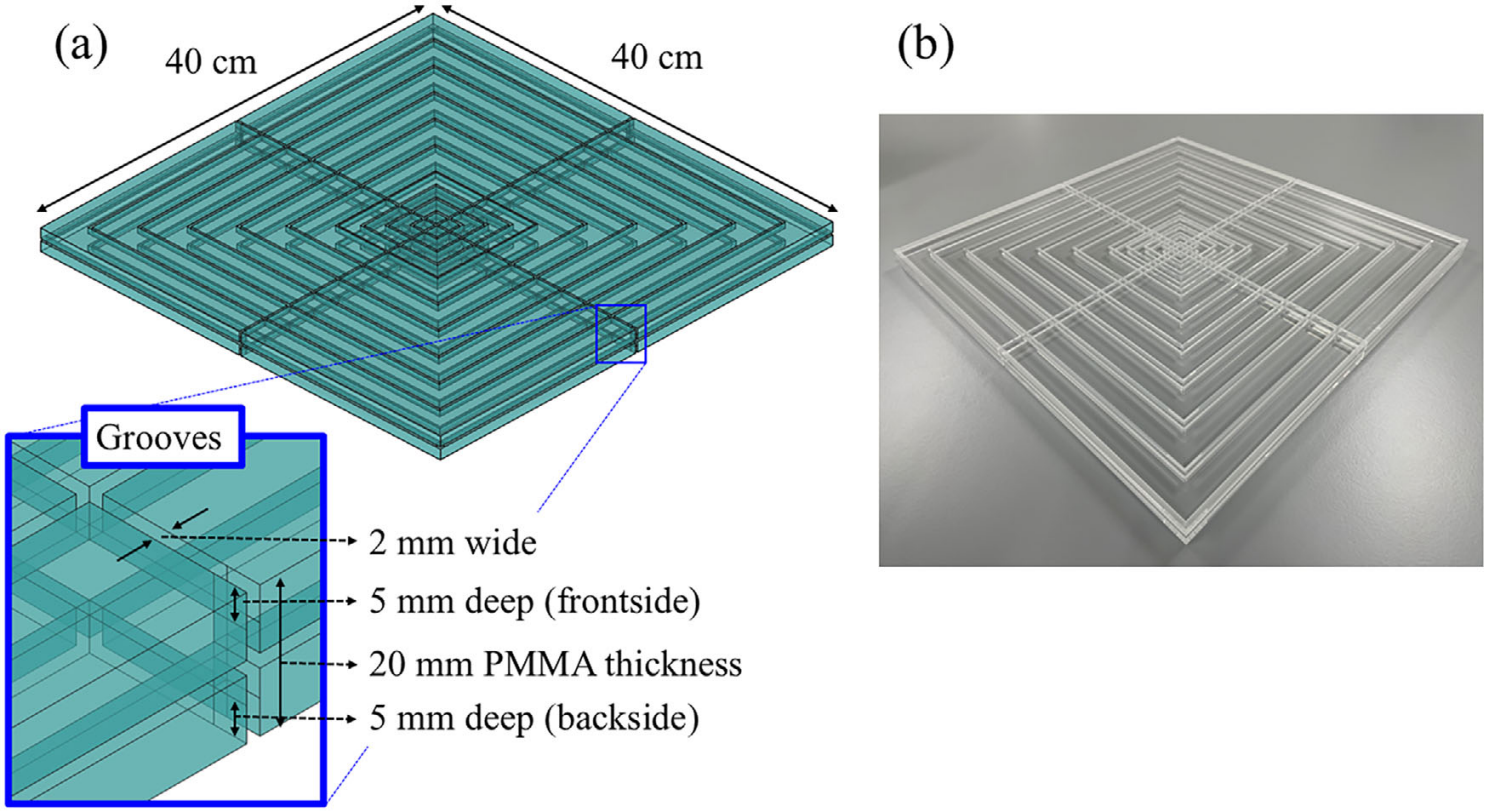
(a) A 40 × 40 × 2 cm^3^ PMMA sizing plate developed to align the X‐ray field size at the PERP. 2‐mm‐wide and 5‐mm‐deep grooves were milled on both sides of a 20‐mm‐thick PMMA plate, with square dimensions of 2.5 × 2.5 cm^2^, 5.0 × 5.0 cm^2^, 7.5 × 7.5 cm^2^, 10 × 10 cm^2^, 15 × 15 cm^2^, 20 × 20 cm^2^, 25 × 25 cm^2^, 30 × 30 cm^2^, and 35 × 35 cm^2^. (b) Photograph of the PMMA sizing plate. PERP, patient entrance reference point; PMMA, polymethyl methacrylate.

A solid‐state dosimeter (AGMS‐DM+, Radcal, Monrovia, CA, USA) was used to measure $K_{a,r}^{AGMS-DM+}$. The solid‐state dosimeter was connected to a laptop through a digitizer and USB cable. The dedicated software (Accu‐Gold 2, Radcal, Monrovia, CA, USA) displayed the $K_{a,r}^{AGMS-DM+}$ values when the AGMS‐DM+ was positioned at the PERP. The dosimeter dimensions were 36 × 20 × 12 mm^3^, with its reference point located 3 mm from the top surface (9 mm from the bottom). Additionally, the AGMS‐DM+ was calibrated within one year of data acquisition using a tube voltage/quality index of 50 kV/0.6, 67 kV/0.6, 83 kV/0.6, and 100 kV/0.6. Its energy dependence was within 1.0% across the entire X‐ray energy range used.

A head phantom (Angiographic CT Head Phantom ACS 41309‐200, Kyoto Kagaku Co., Ltd., Kyoto, Japan) was used to model the outer skin contour, skull, neck vertebrae, and left cerebral arteries. This life‐sized adult phantom is made of urethane‐ and epoxy‐based resins and features contrast‐enhanced left cerebral arteries three‐dimensionally embedded within the brain.^13^

### 2.2 Measurement accuracy of $K_{a,r}^{KAP,Hybrid}$ and $K_{a,r}^{KAP,Meas.}$ relative to $K_{a,r}^{AGMS-DM+}$ as functions of tube voltage, spectral shaping filters, equalization filters, and X‐ray field size at the PERP

Figure 2 shows the measurement setup for $K_{a,r}^{KAP,Hybrid},$ $K_{a,r}^{KAP,Meas.}$, and $K_{a,r}^{AGMS-DM+}$. The X‐ray tube was positioned above the examination table, and the source–to–image receptor distance (SID) was set to 93 cm. The PMMA sizing plate was placed on a 40 × 40 × 20 cm^3^ PMMA block on the examination table. The distance from the source to the mid‐depth of the PMMA sizing plate (SPsD) was set to 56 cm, corresponding to the SPD in the fluoroscopic system (Figure 2a). Additionally, the PMMA sizing plate was aligned with the central axis of the X‐ray beam. The X‐ray field size at the PERP was adjusted, via manual collimation, to 2.5 × 2.5 cm^2^, 5.0 × 5.0 cm^2^, 7.5 × 7.5 cm^2^, 10 × 10 cm^2^, 15 × 15 cm^2^, and 20 × 20 cm^2^ using fluoroscopy. Once the X‐ray field at the PERP was determined, the PMMA sizing plate was replaced with the solid‐state dosimeter. Figure 2b shows the alternative measurement setup for $K_{a,r}^{KAP,Hybrid}$ and $K_{a,r}^{KAP,Meas.}$ where the solid‐state dosimeter was placed on the 20‐cm‐thick PMMA block. When the PMMA sizing plate was located at an SPsD of 70 cm, the X‐ray field size at the PERP could be calculated by multiplying by a factor of 0.8 (= 56 cm / 70 cm). The X‐ray field size at the PERP was then readjusted to 2.0 × 2.0 cm^2^, 4.0 × 4.0 cm^2^, 6.0 × 6.0 cm^2^, 8.0 × 8.0 cm^2^, 12 × 12 cm^2^, and 16 × 16 cm^2^. With this defined geometry, $P_{KA}$, $K_{a,r}^{KAP,Meas.}$, and $K_{a,r}^{AGMS-DM+}$ were measured with the built‐in KAP meter and solid‐state dosimeter over 1 min in fluoroscopy. Subsequently, $K_{a,r}^{KAP,Hybrid}$ was calculated using Equation (1).

**FIGURE 2:**
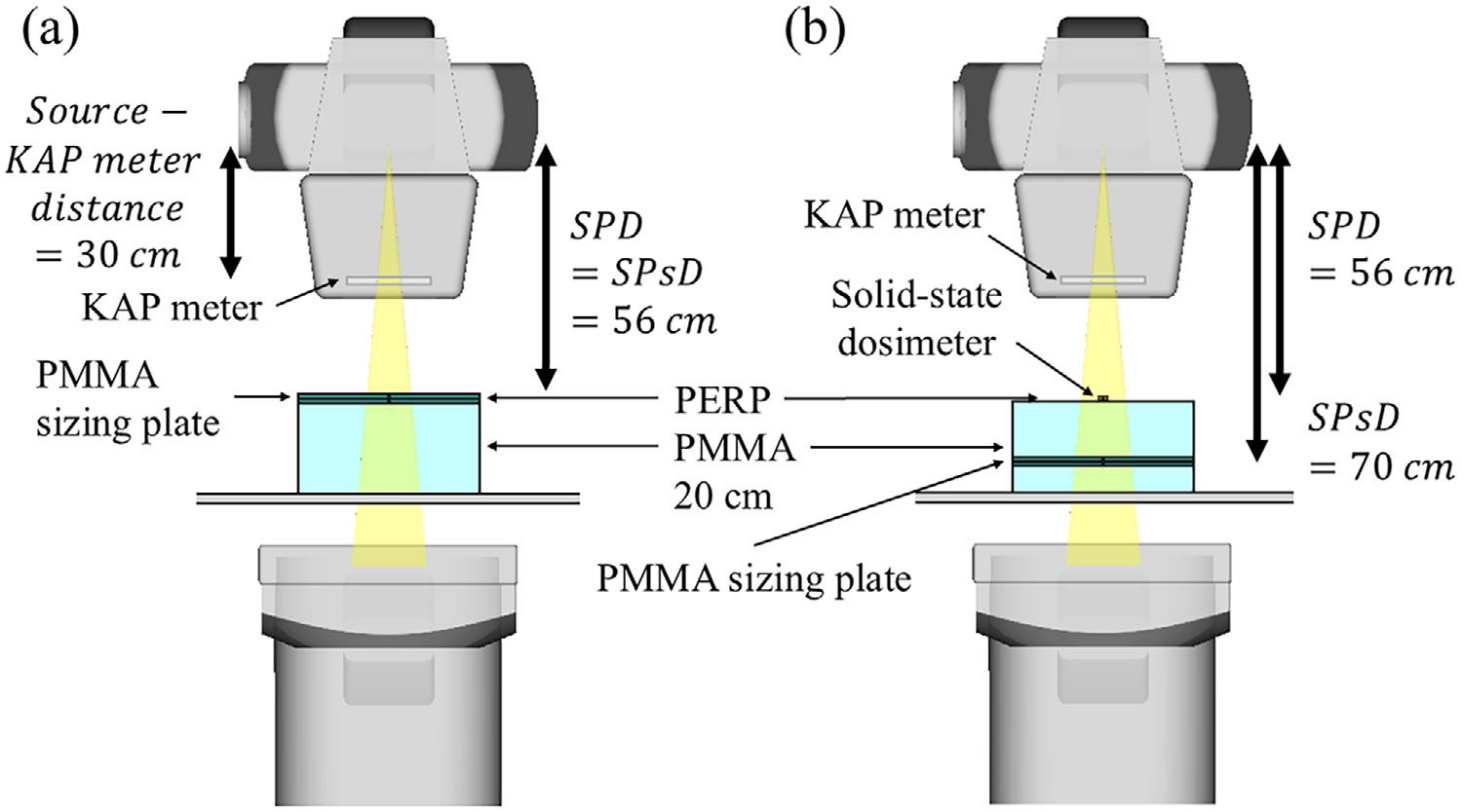
Measurement setup for evaluating $K_{a,r}^{KAP,Hybrid}$, $K_{a,r}^{KAP,Meas.}$, and $K_{a,r}^{AGMS-DM+}$ as functions of tube voltage, spectral shaping filters, equalization filters, and X‐ray field size at the PERP. (a) Configuration where the PMMA sizing plate is aligned with the PERP at the SPD. The X‐ray field size at the PERP can be adjusted to match the size of the milled square on the PMMA sizing plate. (b) Configuration where the PMMA sizing plate is placed at an SPsD of 70 cm. The X‐ray field at the PERP is scaled by a factor of 0.8 (= 56 cm / 70 cm). Abbreviations: $K_{a,r}^{AGMS-DM+}$, reference air kerma measured with AGMS‐DM+; $K_{a,r}^{KAP,Hybrid}$, reference air kerma calculated from the measured $P_{KA}$ combined with collimator‐driven area calculation; $K_{a,r}^{KAP,Meas.}$, directly measured reference air kerma with a small ionization chamber located at the center of the KAP meter; $P_{KA}$, KAP; PERP, patient entrance reference point; PMMA, polymethyl methacrylate, SPD, source–to–PERP distance; SPsD source–to–PMMA sizing plate distance.

Two investigations were performed with the above two geometries. First, the effects of tube voltage and SSFs (1.2 mm Al, 0.03 mm Ta, and 0.2 mm Cu) were evaluated with and without equalization filters. $K_{a,r}^{KAP,Hybrid}$, $K_{a,r}^{KAP,Meas.}$, and $K_{a,r}^{AGMS-DM+}$ were measured at tube voltages of 50, 60, 70, 80, 90, 100, 110, and 115 kV in conjunction with the SSFs of 1.2 mm Al, 0.03 mm Ta, and 0.2 mm Cu, with the remaining imaging parameters fixed at a tube current of 20 mA, a pulse width of 5.0 ms, a frame rate of 7.5 frames per second, and an X‐ray field size of 10 × 10 cm^2^ at the PERP. In interventional neuroradiological procedures, tube voltages commonly range from 60–110 kV.^14^, ^15^ Therefore, this study covered the entire typical clinical range, while also investigating lower (50 kV) and higher (115 kV) boundary values to comprehensively evaluate the energy dependence. In addition, because equalization filters have traditionally been employed to visualize cerebral angiograms clearly and reduce the radiation dose to the patient's eyes,^16^ the same measurements were performed when the right and left equalization filters were inserted symmetrically by 2.5 cm from each side at a fixed X‐ray field size of 10 × 10 cm^2^.

The second investigation was designed to clarify the X‐ray field dependence of $K_{a,r}^{KAP,Hybrid}$ and $K_{a,r}^{KAP,Meas.}$. $K_{a,r}^{KAP,Hybrid}$, $K_{a,r}^{KAP,Meas.}$, and $K_{a,r}^{AGMS-DM+}$ were obtained at the 12 X‐ray field sizes specified above. The measurements were performed for each of the three SSF conditions (1.2 mm Al, 0.03 mm Ta, and 0.2 mm Cu), while the remaining imaging parameters were fixed at a tube voltage of 90 kV, a tube current of 20 mA, a pulse width of 5.0 ms, and a frame rate of 7.5 frames per second. Upon completion of data acquisition, the relative errors for $K_{a,r}^{KAP,Hybrid}$ and $K_{a,r}^{KAP,Meas.}$ were calculated using Equation (3), and these errors were plotted as functions of the tube voltage and the X‐ray field size at the PERP:

(3) $$\begin{array}{cc} & {Error}_{PERP}^{K_{a,r}^{KAP,Hybrid\;or\;Meas.}}\\ & =\frac{K_{a,r}^{KAP,Hybrid\;or\;Meas.}-K_{a,r}^{AGMS-DM+}}{K_{a,r}^{AGMS-DM+}}\times 100\left(\%\right) \end{array}$$

Finally, a linear regression model was employed to describe both $Error_{PERP}^{K_{a,r}^{KAP,Hybrid}}$ and $Error_{PERP}^{K_{a,r}^{KAP,Meas.}}$ as a function of tube voltage, across the filtering conditions with and without equalization filters. For the X‐ray field size dependence, the linear regression model was maintained for $Error_{PERP}^{K_{a,r}^{KAP,Hybrid}}$, whereas a combination of three fitting models was employed to interpolate $Error_{PERP}^{K_{a,r}^{KAP,Meas.}}$. The fitting models yielded the following equations, where A represents the X‐ray field size (in cm^2^):

- Logarithmic fitting: $Error_{PERP}^{K_{a,r}^{KAP,Meas.}}=aln\left(A\right)+b$ (for X‐ray field sizes from 4.0–16.0 cm^2^)
- Second‐degree polynomial fitting: $Error_{PERP}^{K_{a,r}^{KAP,Meas.}}=cA^{2}+dA+e$ (for X‐ray field sizes from 16.0–56.3 cm^2^)
- Second‐degree polynomial fitting: $Error_{PERP}^{K_{a,r}^{KAP,Meas.}}=fA^{2}+gA+h$ (for X‐ray field sizes from 56.3–400 cm^2^).

### 2.3 Feasibility simulation of employing $K_{a,r}^{KAP,Hybrid}$ and $K_{a,r}^{KAP,Meas.}$ as surrogates for $K_{a,skin}$ during interventional neuroradiological procedures

The head phantom was scanned using a 128‐slice whole‐body CT system (SOMATOM go.Top, Siemens Healthineers, Erlangen, Germany). The scan parameters were as follows: a tube voltage of 120 kV, an effective tube current‐time product of 221 mAs, a rotation time of 1.00 s, an exposure time of 16.6 s, a spiral pitch factor of 0.55, a total collimation width of 38.4 mm, a volume computed tomography dose index of 41.2 mGy, and a dose length product of 1398 mGy·cm. The CT images were reconstructed with a slice thickness of 1.00 mm, an increment of 0.70 mm, and a kernel of Hr40. These data were transferred to an open‐source workstation (3D Slicer version 5.8.1) to create three stereolithography models of the outer skin contour, skull with neck vertebrae, and left cerebral arteries.^10^ These stereolithography models were imported into FreeCAD (version 0.21.2), a free and open‐source computer‐aided design software package.^17^ Furthermore, the target centers (imaging isocenters) were set at the inferior‐dorsal region of the third ventricle (brain center) for simulated whole‐cerebral angiography, and at the precallosal segment of the anterior cerebral artery for simulated interventional neuroradiological procedures (Figure 3).

**FIGURE 3:**
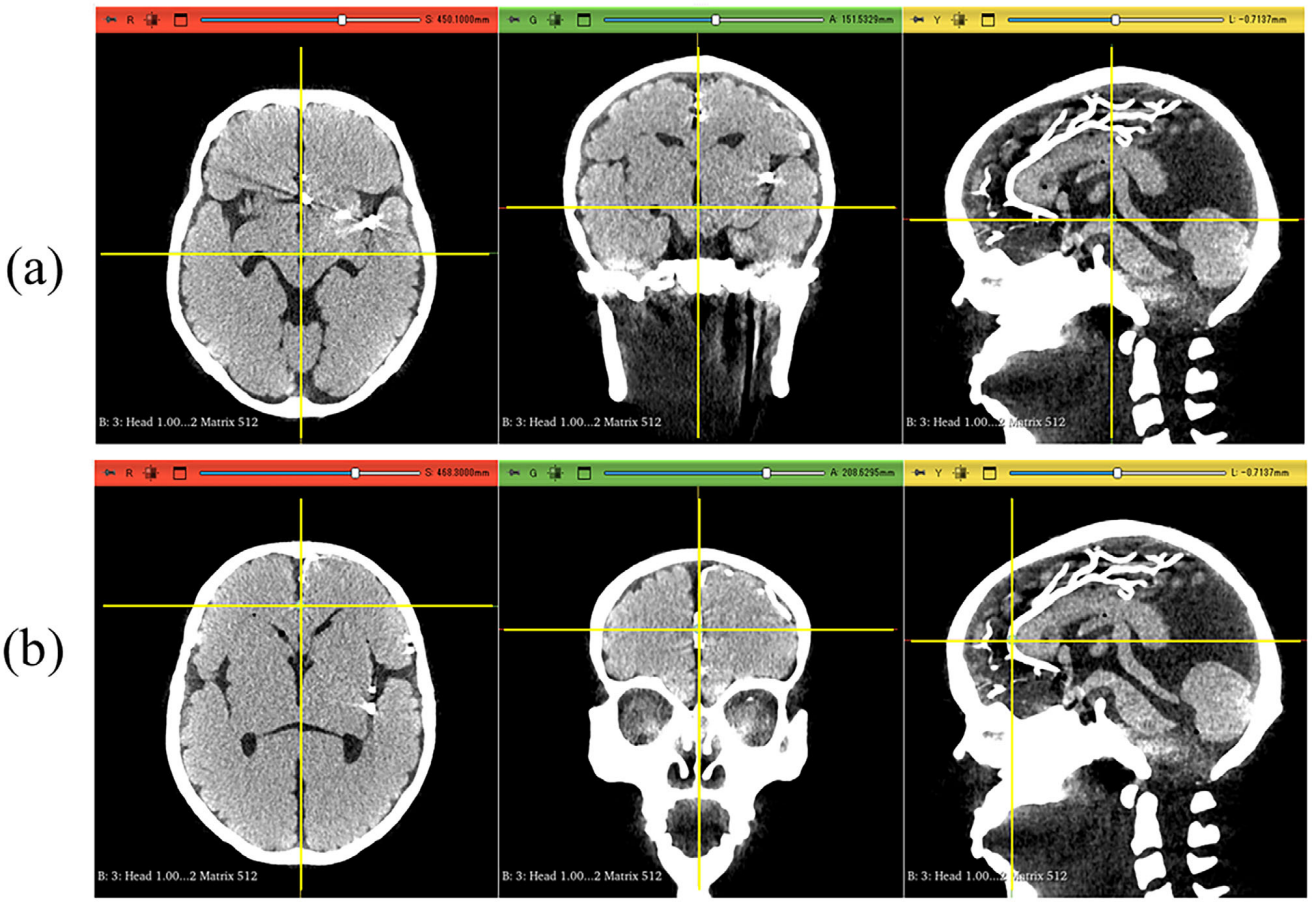
Target centers (imaging isocenters) at (a) the inferior‐dorsal region of the third ventricle (brain center) for whole‐cerebral angiography and (b) the precallosal segment of the anterior cerebral artery for interventional neuroradiological procedures.

The fluoroscopic system was modeled in FreeCAD to emulate the commercially available fluoroscopic system (Ultimax‐i, Canon Medical Systems, Otawara, Japan). The geometrical parameters were as follows: a source–to–axis distance (SAD) of 71 cm, an SPD of 56 cm, a focal spot size with a 1.2‐mm‐diameter circle, and an image receptor cover‐to‐FPD distance of 5 cm. The FOV (X‐ray field size at the FPD) was modeled as 30 × 30 cm^2^ for the simulated whole‐cerebral angiography. Additionally, two FOVs were modeled as 15 × 15 cm^2^ and 3.8 × 3.8 cm^2^ for the simulated interventional neuroradiological procedures.

The entire evaluation was conducted as a geometrical simulation using FreeCAD. The coordinate origin for the x‐, y‐, and z‐axes was set at the isocenter of the simulated C‐arm in FreeCAD. Six typical clinical projections for each FOV were investigated in this virtual environment (Tables 1 and 2). The virtual head phantom was conceptually placed on the examination table, and the two centers of the targets (imaging isocenters) of the phantom were independently aligned with the isocenter of the C‐arm. The SID was determined such that the FPD cover and the phantom surface were separated by 15 cm. Additionally, the SSD was calculated as the distance from the X‐ray focus to the actual skin surface along the central beam axis. The X‐ray field at the PERP was calculated as the area of the X‐ray beam in a plane perpendicular to the beam axis at the PERP using Boolean intersection operations between the beam geometry and the defined plane, followed by numerical area computation. Following the completion of this simulation‐based data generation, to evaluate $K_{a,r}^{KAP,Hybrid}$ and $K_{a,r}^{KAP,Meas.}$ during simulated procedures, $Error_{PERP}^{K_{a,r}^{KAP,Hybrid}}$ and $Error_{PERP}^{K_{a,r}^{KAP,Meas.}}$ obtained in Section 2.2 were first pooled across the three SSFs as a function of X‐ray field size. This pooled error relationship was then applied to the simulated X‐ray field size at the PERP to calculate the values of $K_{a,r}^{KAP,Hybrid}$ and $K_{a,r}^{KAP,Meas.}$ per unit of $K_{a,r}^{AGMS-DM+}$. Furthermore, $K_{a,skin}$ per unit of $K_{a,r}^{AGMS-DM+}$ during the whole cerebral angiography and interventional neuroradiological procedures was derived using Equation (2), along with the X‐ray field size at the PERP and the SSD determined in this section. The derived $K_{a,r}^{KAP,Hybrid}$ and $K_{a,r}^{KAP,Meas.}$ values were compared with $K_{a,skin}$ by calculating the mean relative error at the skin surface ($Error_{skin}^{K_{a,r}^{KAP,Hybrid\;or\;Meas.}}$) using the following equation:

(4) $$\begin{array}{cc} & {Error}_{skin}^{K_{a,r}^{KAP,Hybrid\;or\;Meas.}}\\ & =\frac{K_{a,r}^{KAP,Hybrid\;or\;Meas.}-K_{a,skin}}{K_{a,skin}}\times 100\left(\%\right) \end{array}$$

**TABLE 1:** Geometric data and $K_{a,r}$ errors for routine X‐ray views for the whole cerebral angiography with the isocenter positioned at the center of the brain.

							$Error_{PERP}^{K_{a,r}^{KAP,Hybrid}}$	$Error_{PERP}^{K_{a,r}^{KAP,Meas.}}$		$Error_{skin}^{K_{a,r}^{KAP,Hybrid}}$		$Error_{skin}^{K_{a,r}^{KAP,Meas.}}$	
No	X‐ray tube angulation (°)	FOV (cm)	SID (cm)	SSD (cm)	$SPD^{2}/SSD^{2}$	X‐ray field size at the PERP(cm^2^)	Without correction	Without correction	X‐ray field correction	Without correction	aPERP correction	Without correction	X‐ray field + aPERP corrections
1	0° (PA)	30 × 30	100.0	60.1	0.87	273.7	−4.3%	17.7%	−0.4%	10.3%	−4.3%	35.6%	−0.4%
2	90° (Left lateral)		98.2	61.9	0.82	282.9	−4.6%	18.4%	−0.4%	16.6%	−4.6%	44.6%	−0.4%
3	CRA30° (Towne view)		99.7	59.9	0.87	275.1	−4.3%	17.8%	−0.4%	9.5%	−4.3%	34.8%	−0.4%
4	CAU45° (Waters view)		102.3	60.6	0.85	261.8	−3.9%	16.8%	−0.5%	12.6%	−3.9%	36.7%	−0.5%
5	RAO45°		99.4	60.7	0.85	276.7	−4.4%	17.9%	−0.4%	12.4%	−4.4%	38.5%	−0.4%
6	LAO45°		99.2	60.7	0.85	277.9	−4.4%	18.0%	−0.4%	12.3%	−4.4%	38.7%	−0.4%

Abbreviations: aPERP, alternative patient entrance reference point; CAU, caudal; CRA, cranial; FOV, field of view; $K_{a,r}^{KAP,Hybrid}$, reference air kerma calculated from the measured $P_{KA}$ combined with collimator‐driven area calculation; $K_{a,r}^{KAP,Meas.}$, directly measured reference air kerma with a small ionization chamber located at the center of the kerma‐area product meter; KAP or $P_{KA}$, kerma area product; LAO, left anterior oblique; PA, posteroanterior; PERP, patient entrance reference point; RAO, right anterior oblique; SID, source‐to‐image receptor distance; SPD, source‐to‐PERP distance; SSD, source‐to‐skin distance.

**TABLE 2:** Geometric data and $K_{a,r}$ errors for the third segment of the anterior cerebral artery across 12 X‐ray views in interventional neuroradiological procedures with the isocenter positioned at the precallosal segment.

							$Error_{PERP}^{K_{a,r}^{KAP,Hybrid}}$	$Error_{PERP}^{K_{a,r}^{KAP,Meas.}}$		$Error_{skin}^{K_{a,r}^{KAP,Hybrid}}$		$Error_{skin}^{K_{a,r}^{KAP,Meas.}}$	
No	X‐ray tube angulation (°)	FOV (cm)	SID (cm)	SSD (cm)	$SPD^{2}/SSD^{2}$	X‐ray field size at the PERP (cm^2^)	Without correction	Without correction	X‐ray field correction	Without correction	aPERP correction	Without Correction	X‐ray field + aPERP corrections
1	0° (PA)	15 × 15	94.2	54.8	1.04	77.3	4.6%	−3.0%	0.4%	0.1%	4.6%	−7.1%	0.4%
2	90° (Left lateral)		96.9	63.1	0.79	73.0	4.8%	−3.6%	0.5%	33.1%	4.8%	22.5%	0.5%
3	CRA30° (Towne view)		94.0	53.9	1.08	77.5	4.6%	−2.9%	0.4%	−3.1%	4.6%	−10.1%	0.4%
4	CAU45° (Waters view)		95.7	60.0	0.87	74.9	4.7%	−3.3%	0.4%	20.2%	4.7%	11.0%	0.4%
5	RAO45°		95.0	58.5	0.92	75.9	4.7%	−3.2%	0.4%	14.2%	4.7%	5.7%	0.4%
6	LAO45°		95.0	58.5	0.92	75.8	4.7%	−3.2%	0.4%	14.2%	4.7%	5.7%	0.4%
7	0° (PA)	3.8 × 3.8	94.2	54.8	1.04	5.0	9.1%	−50.2%	−1.2%	4.5%	9.1%	−52.3%	−1.2%
8	90° (Left lateral)		96.9	63.1	0.79	4.8	9.2%	−51.6%	−2.7%	38.6%	9.2%	−38.6%	−2.7%
9	CRA30° (Towne view)		94.0	53.9	1.08	5.1	9.1%	−50.2%	−1.2%	1.1%	9.1%	−53.8%	−1.2%
10	CAU45° (Waters view)		95.7	60.0	0.87	4.9	9.2%	−51.0%	−2.0%	25.3%	9.2%	−43.8%	−2.0%
11	RAO45°		95.0	58.5	0.92	5.0	9.2%	−50.7%	−1.7%	19.1%	9.2%	−46.1%	−1.7%
12	LAO45°		95.0	58.5	0.92	4.9	9.2%	−50.7%	−1.7%	19.1%	9.2%	−46.2%	−1.7%

Abbreviations: aPERP, alternative patient entrance reference point; CAU, caudal; CRA, cranial; FOV, field of view; $K_{a,r}^{KAP,Hybrid}$, reference air kerma calculated from the measured $P_{KA}$ combined with collimator‐driven area calculation; $K_{a,r}^{KAP,Meas.}$, directly measured reference air kerma with a small ionization chamber located at the center of the kerma‐area product meter; KAP or $P_{KA}$, kerma area product; LAO, left anterior oblique; PA, posteroanterior; PERP, patient entrance reference point; RAO, right anterior oblique; SID, source‐to‐image receptor distance; SPD, source‐to‐PERP distance; SSD, source‐to‐skin distance.

Finally, we investigated the effects of the corrections using the X‐ray field size measured by $P_{KA}$/$K_{a,r}^{KAP,Meas.}$ and an alternative PERP (aPERP) positioned 11 cm from the isocenter toward the X‐ray focus.

## 3 RESULTS

Figures 4a–c shows the relative $Error_{PERP}^{K_{a,r}^{KAP,Hybrid}}$ and $Error_{PERP}^{K_{a,r}^{KAP,Meas.}}$ as a function of tube voltage across the three SSFs, and Table 3 shows the linear fitting equations for these relationships. These errors remained relatively constant, ranging from −4.0%–1.9%. When equalization filters were applied, $Error_{PERP}^{K_{a,r}^{KAP,Hybrid}}$ ranged from −49.0%–−33.9%, exceeding the acceptable accuracy tolerance of the KAP meter (± 35%), whereas $Error_{PERP}^{K_{a,r}^{KAP,Meas,}}$ (−15.8%–−10.8%) remained within the tolerance. Figures 5a–c show the relative $Error_{PERP}^{K_{a,r}^{KAP,Hybrid}}$ and $Error_{PERP}^{K_{a,r}^{KAP,Meas.}}$ as a function of X‐ray field size at the PERP, and Table 4 shows the fitting equations for these relationships. $Error_{PERP}^{K_{a,r}^{KAP,Hybrid}}$ decreased linearly from 12.7%–−9.7% with increasing X‐ray field size at the PERP. In contrast, $Error_{PERP}^{K_{a,r}^{KAP,Meas.}}$ was larger than $Error_{PERP}^{K_{a,r}^{KAP,Hybrid}}$ and dropped sharply at an X‐ray field size below 100 cm^2^. Beyond 100 cm^2^, $Error_{PERP}^{K_{a,r}^{KAP,Meas.}}$ increased with increasing X‐ray field size, ranging from −58.3%–26.1%.

**FIGURE 4:**
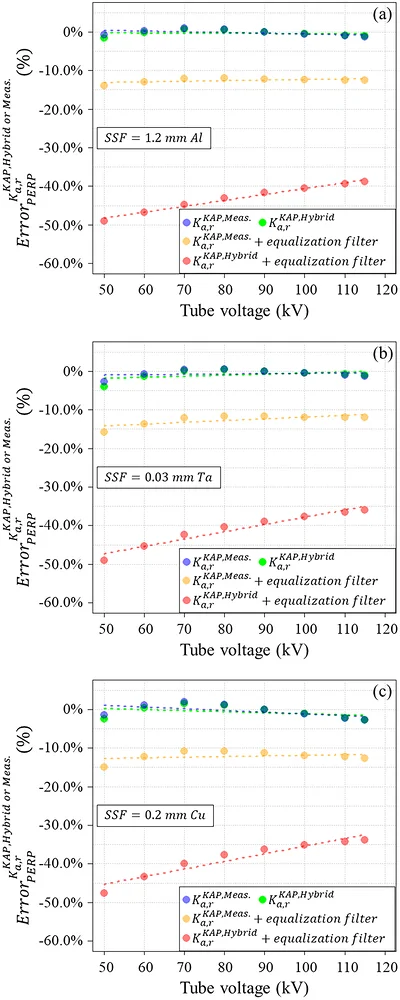
A–C $Error_{PERP}^{K_{a,r}^{KAP,Hybrid}}$ and $Error_{PERP}^{K_{a,r}^{KAP,Meas.}}$ for SSFs of (a) 1.2 mm Al, (b) 0.03 mm Ta, and (c) 0.2 mm Cu plotted as a function of tube voltage with and without the equalization filters. $K_{a,r}^{KAP,Hybrid}$, reference air kerma calculated from the measured $P_{KA}$ combined with collimator‐driven area calculation; $K_{a,r}^{KAP,Meas.}$, directly measured reference air kerma with a small ionization chamber located at the center of the kerma‐area product meter; $P_{KA}$, kerma‐area product; PERP, patient entrance reference point; SSF, spectral shaping filter.

**TABLE 3:** Linear fitting equations for $Error_{PERP}^{K_{a,r}^{KAP,Hybrid}}$ and $Error_{PERP}^{K_{a,r}^{KAP,Meas.}}$ of SSFs with 1.2 mm Al, 0.03 mm Ta, and 0.2 mm Cu as a function of tube voltage, with and without equalization filters (corresponding to Figures 4a–c).

SSF	$Error_{PERP}^{K_{a,r}^{KAP,Hybrid}}$		$Error_{PERP}^{K_{a,r}^{KAP,Meas.}}$	
	Without equalization filter	With equalization filter	Without equalization filter	With equalization filter
1.2 mm Al	$-4.084\times 10^{-5}V-1.792\times 10^{-4}$	$1.525\times 10^{-3}V-5.586\times 10^{-1}$	$-1.927\times 10^{-4}V+1.401\times 10^{-2}$	$1.451\times 10^{-4}V-1.379\times 10^{-1}$
0.03 mm Ta	$2.726\times 10^{-4}V-3.188\times 10^{-2}$	$1.894\times 10^{-3}V-5.675\times 10^{-1}$	$8.297\times 10^{-5}V-1.375\times 10^{-2}$	$4.534\times 10^{-4}V-1.641\times 10^{-1}$
0.2 mm Cu	$-2.749\times 10^{-4}V+1.632\times 10^{-2}$	$1.976\times 10^{-3}V-5.517\times 10^{-1}$	$-4.512\times 10^{-4}V+3.369\times 10^{-2}$	$1.598\times 10^{-4}V-1.347\times 10^{-1}$

*Note*: V represents the tube voltage in kV.

Abbreviations: $K_{a,r}^{KAP,Hybrid}$, reference air kerma calculated from the measured $P_{KA}$ combined with collimator‐driven area calculation; $K_{a,r}^{KAP,Meas.}$, directly measured reference air kerma with a small ionization chamber located at the center of the kerma‐area product meter; KAP or $P_{KA}$, kerma‐area product; PERP, patient entrance reference point; SSF, spectral shaping filter.

**FIGURE 5:**
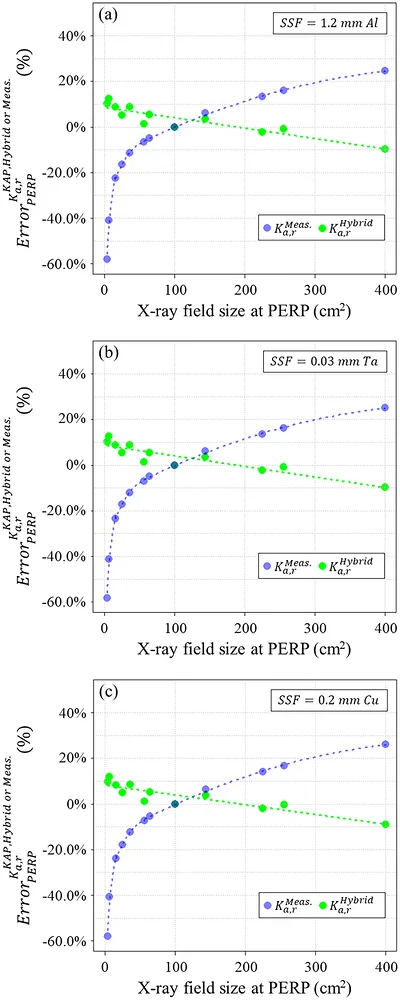
A–C $Error_{PERP}^{K_{a,r}^{KAP,Hybrid}}$ and $Error_{PERP}^{K_{a,r}^{KAP,Meas.}}$ for SSFs of (a) 1.2 mm Al, (b) 0.03 mm Ta, and (c) 0.2 mm Cu plotted as a function of X‐ray field size. $K_{a,r}^{KAP,Hybrid}$, reference air kerma calculated from the measured $P_{KA}$ combined with collimator‐driven area calculation; $K_{a,r}^{KAP,Meas.}$, directly measured reference air kerma with a small ionization chamber located at the center of the kerma‐area product meter; $P_{KA}$, kerma‐area product; PERP, patient entrance reference point; SSF, spectral shaping filter.

**TABLE 4:** Fitting equations for $Error_{PERP}^{K_{a,r}^{KAP,Hybrid}}$ and $Error_{PERP}^{K_{a,r}^{KAP,Meas.}}$ of SSFs with 1.2 mm Al, 0.03 mm Ta, and 0.2 mm Cu as a function of X‐ray field size (corresponding to Figures 5a–c).

	$Error_{PERP}^{K_{a,r}^{KAP,Hybrid}}$	$Error_{PERP}^{K_{a,r}^{KAP,Meas.}}$		
	X‐ray field size (cm^2^)	X‐ray field size (cm^2^)	X‐ray field size (cm^2^)	X‐ray field size (cm^2^)
SSF	4.0–400	4.0–16.0	16.0–56.3	56.3–400
1.2 mm Al	$-4.555\times 10^{-4}A+8.641\times 10^{-2}$	$2.475\times 10^{-1}ln\left(A\right)-8.987\times 10^{-1}$	$-8.114\times 10^{-5}A^{2}+9.741\times 10^{-3}A-3.584\times 10^{-1}$	$-1.605\times 10^{-6}A^{2}+1.622\times 10^{-3}A-1.473\times 10^{-1}$
0.03 mm Ta	$-4.628\times 10^{-4}A+8.747\times 10^{-2}$	$2.421\times 10^{-1}ln\left(A\right)-8.932\times 10^{-1}$	$-8.249\times 10^{-5}A^{2}+1.001\times 10^{-2}A-3.724\times 10^{-1}$	$-1.665\times 10^{-6}A^{2}+1.670\times 10^{-3}A-1.522\times 10^{-1}$
0.2 mm Cu	$-4.280\times 10^{-4}A+8.269\times 10^{-2}$	$2.358\times 10^{-1}ln\left(A\right)-8.798\times 10^{-1}$	$-8.136\times 10^{-5}A^{2}+9.991\times 10^{-3}A-3.777\times 10^{-1}$	$-1.727\times 10^{-6}A^{2}+1.736\times 10^{-3}A-1.587\times 10^{-1}$

*Note*: A represents the X‐ray field size in cm^2^.

Abbreviations: $K_{a,r}^{KAP,Hybrid}$, reference air kerma calculated from the measured $P_{KA}$ combined with collimator‐driven area calculation; $K_{a,r}^{KAP,Meas.}$, directly measured reference air kerma with a small ionization chamber located at the center of the kerma‐area product meter; KAP or $P_{KA}$, kerma‐area product; PERP, patient entrance reference point; SSF, spectral shaping filter.

Figure 6 shows a lateral view of the simulated whole cerebral angiography modeled in FreeCAD. Because the *SSD* was 61.9 cm, the $SPD^{2}/SSD^{2}$ was 0.82, indicating that $K_{a,skin}$ was reduced to 82% of $K_{a,r}$. Table 1 shows the results of the routine six X‐ray views in the whole cerebral angiography. The mean ± standard deviation (SD) of the SID and SSD were 99.8 ± 1.4 cm and 60.7 ± 0.7 cm, respectively. Therefore, the mean ± SD of the $SPD^{2}/SSD^{2}$ for the whole cerebral angiography was 0.85 ± 0.02. Furthermore, the means ± SDs of $Error_{PERP}^{K_{a,r}^{KAP,Hybrid}}$ and $Error_{PERP}^{K_{a,r}^{KAP,Meas.}}$ were −4.3% ± 0.2% and 17.8% ± 0.5%, respectively. Notably, $K_{a,r}^{KAP,Hybrid}$ underestimated $K_{a,r}^{AGMS-DM+}$ in the whole cerebral angiography, although $Error_{PERP}^{K_{a,r}^{KAP,Hybrid}}$ was within the International Electrotechnical Commission specified tolerance (± 35%) for KAP meter accuracy (hereafter, “the specified tolerance”).^18^ Furthermore, the means ± SDs of $Error_{skin}^{K_{a,r}^{KAP,Hybrid}}$ and $Error_{skin}^{K_{a,r}^{KAP,Meas.}}$ were 12.3% ± 2.5% and 38.1% ± 3.5%, respectively. These findings indicate that $K_{a,r}^{KAP,Meas.}$ should be employed to monitor $K_{a,skin}$ with appropriate X‐ray field size and aPERP corrections for the whole cerebral angiography. $Error_{skin}^{K_{a,r}^{KAP,Hybrid}}$ was reduced with the aPERP correction, yielding a mean ± SD of −4.3% ± 0.2%, whereas $Error_{skin}^{K_{a,r}^{KAP,Meas.}}$ was reduced with both the X‐ray field size and aPERP corrections, yielding a mean ± SD of −0.4% ± 0.0%.

**FIGURE 6:**
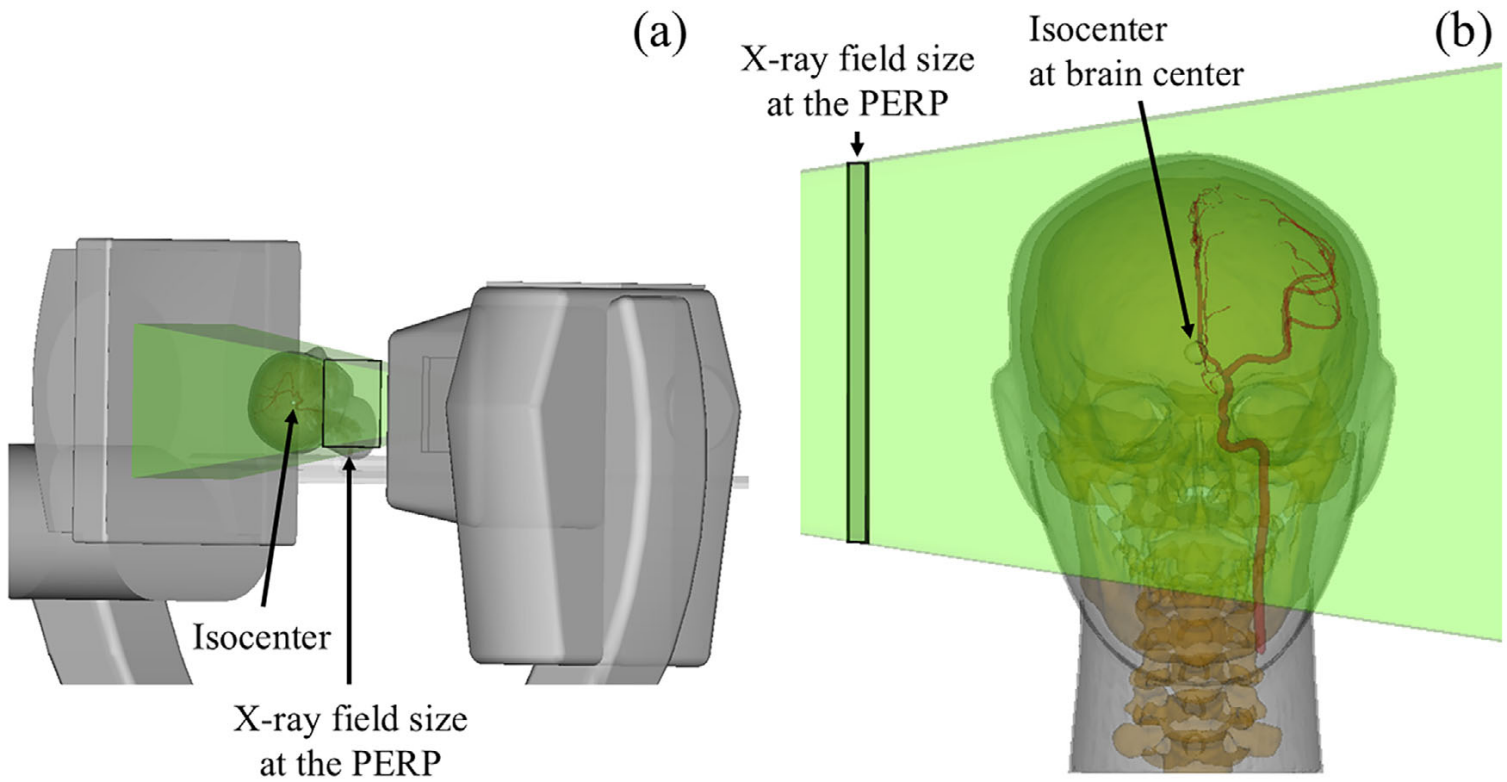
Lateral view of the simulated whole cerebral angiography modeled in FreeCAD. (a) Overview and (b) enlarged top view. Because the SSD was 61.9 cm, the $SPD^{2}/SSD^{2}$ ratio was 0.82, indicating that $K_{a,skin}$ was reduced to 82% of $K_{a,r}$. $K_{a,r}$, reference air kerma; $K_{a,skin}$, incident air kerma at the skin; PERP, patient entrance reference point; SPD, source–to–PERP distance; SSD, source–to–skin distance.

Figure 7 shows a posteroanterior view of the simulated precallosal segment of the anterior cerebral artery for interventional neuroradiological procedures modeled in FreeCAD. Because the *SSD* was 54.8 cm, the $SPD^{2}/SSD^{2}$ ratio was 1.04, indicating that $K_{a,skin}$ increased by 4% relative to $K_{a,r}$. Table 2 shows the results of the 12 X‐ray views across two FOVs for the precallosal segment of the anterior cerebral artery in the interventional neuroradiological procedures. The means ± SDs of the SID and SSD were 95.1 ± 1.0 cm and 58.1 ± 3.2 cm, respectively. Therefore, the mean ± SD of the $SPD^{2}/SSD^{2}$ ratio was 0.94 ± 0.10. Furthermore, the means ± SDs of $Error_{PERP}^{K_{a,r}^{KAP,Hybrid}}$ were 4.7% ± 0.1% and 9.2% ± 0.0% for the 15 × 15 cm^2^ and 3.8 × 3.8 cm^2^ FOVs, respectively, whereas those of $Error_{PERP}^{K_{a,r}^{KAP,Meas.}}$ were −3.2% ± 0.2% and −50.7% ± 0.5%. These findings indicate that $K_{a,r}^{KAP,Hybrid}$ can be used to monitor $K_{a,r}$. However, $K_{a,r}^{KAP,Meas.}$ at small fields underestimated $K_{a,r}$, exceeding the specified tolerance.^18^ Because $K_{a,r}^{KAP,Meas.}$ is influenced by the X‐ray field size (Figure 5), the underestimation is unavoidable when the X‐ray field size at the PERP is less than 100 cm^2^. Similarly, the means ± SDs of $Error_{skin}^{K_{a,r}^{KAP,Hybrid}}$ were 13.1% ± 13.3% and 18.0% ± 13.8% for the 15 × 15 cm^2^ and 3.8 × 3.8 cm^2^ FOVs, respectively, whereas those of $Error_{skin}^{K_{a,r}^{KAP,Meas.}}$ were 4.6% ± 11.9% and −46.8% ± 5.6%. On the other hand, $Error_{skin}^{K_{a,r}^{KAP,Hybrid}}$ was reduced with the aPERP correction, yielding means and SDs of 4.7% ± 0.1% and 9.2% ± 0.0% for the 15 × 15 cm^2^ and 3.8 × 3.8 cm^2^ FOVs, respectively, whereas $Error_{skin}^{K_{a,r}^{KAP,Meas.}}$ was reduced with both X‐ray field size and aPERP corrections, yielding means and SDs of 0.4% ± 0.0% and −1.8% ± 0.6% for the 15 × 15 cm^2^ and 3.8 × 3.8 cm^2^ FOVs, respectively.

**FIGURE 7:**
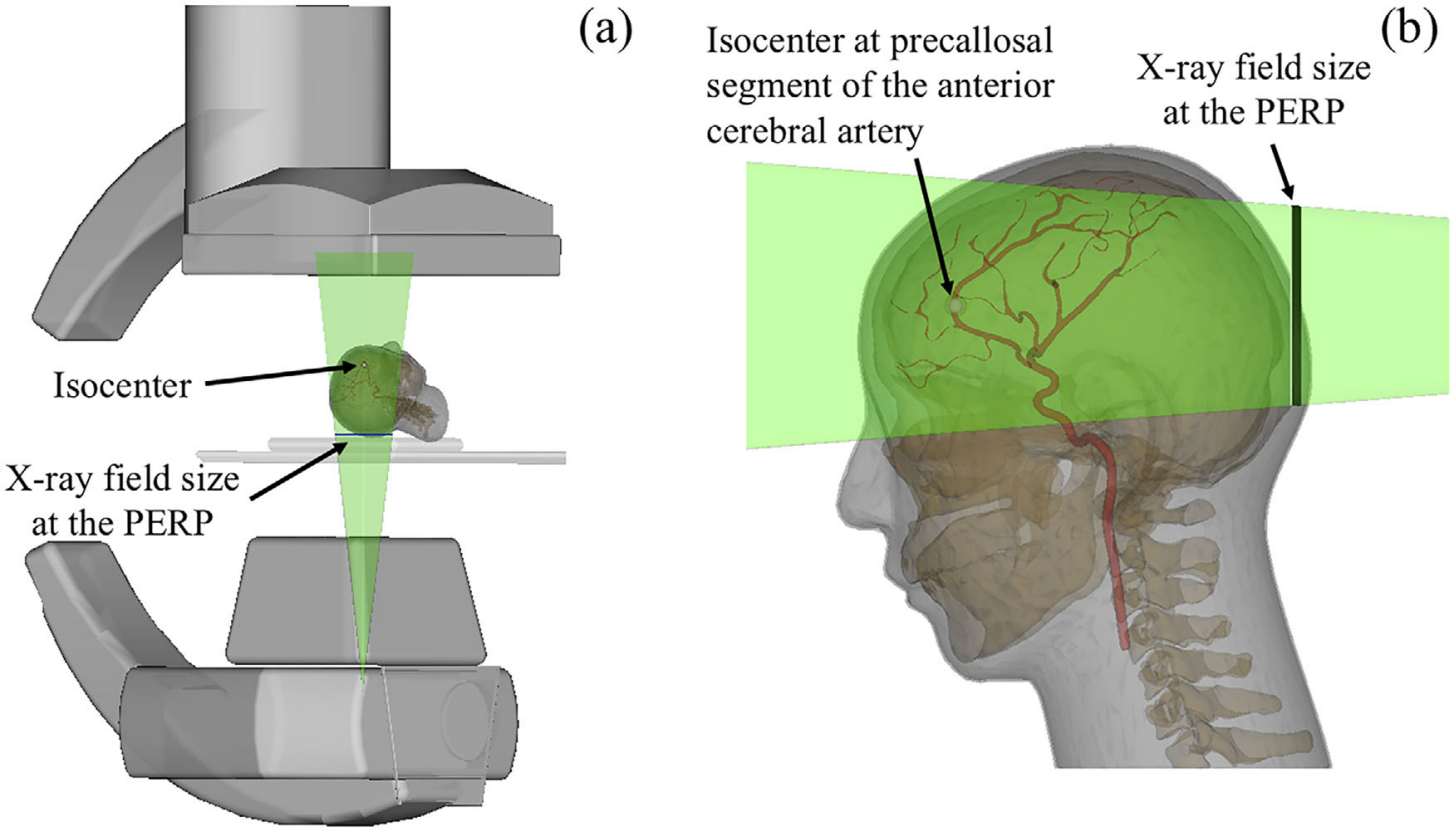
Posteroanterior view of the simulated precallosal segment of the anterior cerebral artery for interventional neuroradiological procedures modeled in FreeCAD. (a) Overview and (b) enlarged lateral view. Because the SSD was 54.8 cm, the $SPD^{2}/SSD^{2}$ ratio was 1.04, indicating that $K_{a,skin}$ increased by 4% relative to $K_{a,r}$. $K_{a,r}$, reference air kerma; $K_{a,skin}$, incident air kerma at the skin; PERP, patient entrance reference point; SPD, source–to–PERP distance; SSD, source–to–skin distance.

## 4 DISCUSSION

This study investigated the accuracy of $K_{a,r}^{KAP,Hybrid}$ and $K_{a,r}^{KAP,Meas.}$ as functions of tube voltage across the three SSFs with and without equalization filters. Although these errors remained relatively constant, applying the equalization filters caused $Error_{PERP}^{K_{a,r}^{KAP,Hybrid}}$ to exceed the specified tolerance. Furthermore, the accuracy of both metrics was investigated as a function of X‐ray field size across the three SSFs. $Error_{PERP}^{K_{a,r}^{KAP,Hybrid}}$ was within the specified tolerance, whereas $Error_{PERP}^{K_{a,r}^{KAP,Meas.}}$ dropped sharply at a field size below 100 cm^2^ and exceeded the specified tolerance. While most $Error_{skin}^{K_{a,r}^{KAP,Hybrid}}$ and $Error_{skin}^{K_{a,r}^{KAP,Meas.}}$ values remained within the ± 35% tolerance during the simulated interventional neuroradiological procedures, $Error_{skin}^{K_{a,r}^{KAP,Meas.}}$ at the 3.8 × 3.8 cm^2^ FOV exceeded it. However, $Error_{skin}^{K_{a,r}^{KAP,Meas.}}$ at this FOV was reduced by applying the X‐ray field size and aPERP corrections, yielding a mean and SD of −1.8% ± 0.6%. These findings indicate that applying equalization filters in clinical settings may cause $K_{a,r}^{KAP,Hybrid}$ to underestimate $K_{a,r}$ beyond the specified tolerance. In contrast, $K_{a,r}^{KAP,Meas.}$ must be employed to monitor $K_{a,skin}$ with appropriate X‐ray field size and aPERP corrections during interventional neuroradiological procedures.

In this study, the solid‐state dosimeter was employed to measure $K_{a,r}^{AGMS-DM+}$ in place of an ionization chamber. The backscattered radiation from the PMMA plastic was not included in $K_{a,r}^{KAP,Hybrid}$, $K_{a,r}^{KAP,Meas.}$, and $K_{a,r}^{AGMS-DM+}$. Because the solid‐state dosimeter is manufactured with a tin, tungsten, or lead backing, $K_{a,r}^{AGMS-DM+}$ can be directly measured even when the solid‐state dosimeter is placed on the PMMA plastic.^12^

$Error_{PERP}^{K_{a,r}^{KAP,Hybrid}}$ and $Error_{PERP}^{K_{a,r}^{KAP,Meas.}}$ values were within ± 4.0% as a function of tube voltage across the three SSFs, demonstrating no appreciable impact on the KAP meter employed. This is because the KAP meter is an ionization chamber structurally designed to be less susceptible to the effects of X‐ray energy. However, when equalization filters were applied, $Error_{PERP}^{K_{a,r}^{KAP,Hybrid}}$ ranged from −49.0%–−33.9%, exceeding the specified tolerance, whereas $Error_{PERP}^{K_{a,r}^{KAP,Meas.}}$ (−15.8%–−10.8%) remained within the tolerance. ^18^ Because the flatness of the X‐ray intensity passing through the KAP meter was decreased by the equalization filter, $P_{KA}$ decreases even if $K_{a,r}$ is identical.^19^ As previously described in Equation (1), because $K_{a,r}^{KAP,Hybrid}$ is calculated as $P_{KA}/X-ray\;field\;size$, $K_{a,r}^{KAP,Hybrid}$ underestimates $K_{a,r}$. $Error_{PERP}^{K_{a,r}^{KAP,Hybrid}}$ also decreased linearly from 12.7%–−9.7% with increasing X‐ray field size at the PERP. Although $P_{KA}$ is measured through a single large‐area parallel‐plate ionization chamber, $P_{KA}$ does not increase proportionally with increasing X‐ray field size at the PERP. This nonlinear proportionality is primarily driven by the physical and geometric characteristics of the heel effect under ultra‐short‐distance conditions, as the KAP meter is positioned extremely close to the X‐ray tube focal spot.^20^ At such close proximity, the large angular spread of the beam exacerbates the nonlinear, asymmetric intensity profile caused by exponential self‐absorption on the deep anode side. As the X‐ray field size at the PERP expands, this inherent nonlinear fluence is integrated across the active area of the KAP meter, causing variations and uncertainties in both $P_{KA}$ and $K_{a,r}^{KAP,Hybrid}$.^20^ While mechanical factors such as collimation or alignment errors may also overlay this phenomenon, the nonlinear physics of the heel effect at large fields remains a primary driver.

Similarly, $Error_{PERP}^{K_{a,r}^{KAP,Meas.}}$ was observed to exceed the specified tolerance when the X‐ray field size at the PERP was less than 5.1 cm^2^ (Table 2). As the X‐ray field size at the PERP is reduced, the collimated X‐ray field at the KAP meter position becomes smaller than the active area of the inner central chamber. The KAP meter was installed 30 cm from the X‐ray tube focus. When the X‐ray field size at the PERP was adjusted to yield an FOV of 2.0 × 2.0 cm^2^, the collimated X‐ray field size at the KAP meter position was 1.1 × 1.1 cm^2^. This field size is smaller than the active area of the inner central chamber (1.2 × 1.2 cm^2^), resulting in under‐irradiation of the sensitive volume. Furthermore, the contributions of off‐focus radiation and scattered radiation inside the KAP meter theoretically increase as a function of the X‐ray field size at the PERP. These composite physical and geometrical factors introduce significant variations and uncertainties into the $K_{a,r}^{KAP,Hybrid}$ and $K_{a,r}^{KAP,Meas.}$ measurements.^21^

$K_{a,r}$ is nearly equal to $K_{a,skin}$ in percutaneous coronary intervention.^10^ However, this difference is larger in the whole cerebral angiography (Table 1). The mean ± SD of the SSD and $SPD^{2}/SSD^{2}$ ratio are 60.7 ± 0.7 cm and 0.85 ± 0.02, respectively, in the whole cerebral angiography. This finding indicates that $K_{a,r}$ may constantly overestimate $K_{a,skin}$ by 15%. Therefore, the aPERP positioned 11 cm from the isocenter toward the X‐ray focus may be useful for the whole cerebral angiography. When the aPERP is applied for distance correction, the mean ± SD of the $SPD^{2}/SSD^{2}$ ratio improves to 0.98 ± 0.02 across all whole cerebral angiography procedures. Therefore, $K_{a,aPERP}$ becomes approximately equal to $K_{a,skin}$. Currently, radiation dose alerting thresholds for peak skin dose follow‐up cases in interventional neuroradiology are often set to a uniform fixed value, such as 5 Gy for the whole cerebral angiography. However, our findings could potentially be applied to establish a dynamic, geometry‐dependent alert system. For instance, by integrating the proposed spatial factors, the clinical alert level could be adjusted dynamically using a threshold formula such as $5\times {\left(SaPD/SSD\right)}^{2}$, where SaPD represents the source‐to‐aPERP distance. Such an advanced monitoring system would allow for more precise, individualized tracking of skin dose risks based on real‐time examination geometries, significantly enhancing proactive radiation protection for patients.

In this study, the difference between the whole cerebral angiography and the interventional neuroradiological procedures was the FOV utilized. Whole cerebral angiography conventionally images the whole brain, necessitating an FOV of 30 × 30 cm^2^. In clinical settings, a small X‐ray field is often employed to image target lesions under magnification geometry to conduct interventional neuroradiological procedures. The X‐ray field size at the PERP for the 15 × 15 cm^2^ FOV was approximately 73 cm^2^ (Table 2). In these X‐ray fields, $Error_{skin}^{K_{a,r}^{KAP,Meas.}}$ was within the specified tolerance. However, recent advancements in the fluoroscopic systems have enabled an FOV under 3.8 × 3.8 cm^2^ for the coiling of intracranial aneurysms.^22^, ^23^ The X‐ray field size at the PERP for the 3.8 × 3.8 cm^2^ FOV was less than 5.1 cm^2^ (Table 2). In these X‐ray fields, the mean $Error_{PERP}^{K_{a,r}^{KAP,Meas.}}$ and $Error_{skin}^{K_{a,r}^{KAP,Meas.}}$ were −50.7% and −46.8%, respectively, which exceeded the specified tolerance.^18^ Furthermore, automatic exposure control conventionally increases the radiation dose while decreasing the FOV to maintain adequate image quality.^24^ In this case, even though the radiation dose is increased, $K_{a,r}^{KAP,Meas.}$ displays half of the actual $K_{a,r}$ and $K_{a,skin}$. Because the chamber‐in‐chamber KAP meter consists of two chambers, the X‐ray field size at the PERP can be measured from $P_{KA}$ and $K_{a,r}^{KAP,Meas.}$ using Equation (1): $X-ray\;field\;size=P_{KA}/K_{a,r}^{KAP,Meas.}$. Therefore, in addition to the application of the aPERP, an automatic correction algorithm for the dependence of $K_{a,r}^{KAP,Meas.}$ on the X‐ray field size at the PERP should be developed to reduce uncertainty and applied in the interventional neuroradiological procedures. In this study, we investigated the effects of the corrections using the X‐ray field size measured by $P_{KA}$/$K_{a,r}^{KAP,Meas.}$ and the aPERP positioned 11 cm from the isocenter toward the X‐ray focus. As a result, $Error_{skin}^{K_{a,r}^{KAP,Hybrid}}$ was reduced with the aPERP correction, yielding means and SDs of −4.3% ± 0.2%, 4.7% ± 0.1%, and 9.2% ± 0.0% for the 30 × 30 cm^2^, 15 × 15 cm^2^, and 3.8 × 3.8 cm^2^ FOVs, respectively, whereas $Error_{skin}^{K_{a,r}^{KAP,Meas.}}$ was reduced with both X‐ray field size and aPERP corrections, yielding means and SDs of −0.4% ± 0.0%, 0.4% ± 0.0%, and −1.8% ± 0.6% for the 30 × 30 cm^2^, 15 × 15 cm^2^, and 3.8 × 3.8 cm^2^ FOVs, respectively.

Skin dose is essential for predicting skin injuries and is calculated using the following equation:

(5) $$Skin\;dose=K_{a,skin}\;BSF\;\frac{{\left[\mu_{en}/\rho \right]}_{skin}}{{\left[\mu_{en}/\rho \right]}_{air}}$$

where BSF, ${\left[\mu_{en}/\rho \right]}_{skin}$, and ${\left[\mu_{en}/\rho \right]}_{air}$ are the backscatter factor and the mass energy‐absorption coefficients for the skin and air, respectively. The BSF for ICRU tissue is in the range of 1.25–1.41 for tube voltages between 50 and 150 kV.^25^ Additionally, the $\frac{{\left[\mu_{en}/\rho \right]}_{skin}}{{\left[\mu_{en}/\rho \right]}_{air}}$ ratio ranges from 1.05–1.09 for the typical radiation beam quality of angiographic systems.^24^, ^26^ The peak skin dose is required to assess the overlap of the X‐ray fields. The overlap of the X‐ray fields on the actual skin surface can be determined through the X‐ray beam modeling based on computer‐aided design employed in this study.^10^

These findings present a clear paradigm for clinical dose monitoring in neuroradiology. While $K_{a,r}^{KAP,Hybrid}$ provides sufficient accuracy for standard, wide‐field diagnostic cerebral angiography, it fails to capture the significant underestimation caused by equalization filters. Conversely, $K_{a,r}^{KAP,Meas.}$, when combined with our proposed X‐ray field size and aPERP corrections, demonstrated superior applicability for highly targeted, small‐field interventional neuroradiology. Clinicians and medical physicists should therefore strategically select the appropriate surrogate metric depending on the specific procedural workflow to ensure accurate skin dose tracking.

This study has four limitations. First, the measurements were performed using a built‐in chamber‐in‐chamber KAP meter consisting of a single large‐area parallel‐plate ionization chamber and a small central chamber. Many angiographic systems are equipped with a logical or physical single chamber rather than a chamber‐in‐chamber KAP meter to monitor $K_{a,r}^{KAP}$ and $P_{KA}$. Therefore, $K_{a,r}^{KAP}$ obtained in this study may differ from that measured using conventional single‐chamber KAP meters. Second, automatic exposure rate control was not experimentally evaluated in this study. However, its general characteristics under the TG‐125 geometry have been thoroughly documented previously, and linearity has been reported as a function of the spectral shaping filter and PMMA thickness.^12^ In addition, the attenuation caused by the table and pad was also excluded from this study. Since various combinations of tables and pads are installed in catheterization laboratories, a universal evaluation of these factors was beyond the scope of this study. Furthermore, in interventional neuroradiological procedures, lateral X‐ray angles are frequently employed, where the X‐ray beam does not pass through the table or pad. Therefore, evaluating the radiation dose independently of these attenuations is crucial for precise radiation safety management. Third, the influence of the skull on backscatter factors was not accounted for in this study. The backscatter factor in the presence of the skull has been reported to decrease by up to 15% compared with that measured for a homogeneous water phantom.^27^ Therefore, incorporating these insights is essential for an accurate evaluation of skin dose. Fourth, the two target centers (imaging isocenters) were set to the inferior‐dorsal region of the third ventricle for the whole cerebral angiography and the precallosal segment of the anterior cerebral artery for the interventional neuroradiological procedures. Therefore, other centers of the targets (imaging isocenters) might be alternative options for assessing the SSD, the $SPD^{2}/SSD^{2}$ ratio, and the appropriate location of the aPERP. However, because most cerebral angiography and interventional neuroradiological procedures are performed by aligning the center of the brain with the imaging isocenter, $K_{a,r}^{KAP,Hybrid}$ and $K_{a,r}^{KAP,Meas.}$ calibrated at an aPERP of 11 cm from the isocenter may be useful for reducing the uncertainty of $K_{a,skin}$.

## 5 CONCLUSION

$K_{a,r}^{KAP,Hybrid}$ can be employed to monitor $K_{a,r}$ and $K_{a,skin}$ within the specified tolerance. However, it should be noted that when the equalization filters were applied, $Error_{PERP}^{K_{a,r}^{KAP,Hybrid}}$ exceeded the specified tolerance. In contrast, $Error_{PERP}^{K_{a,r}^{KAP,Meas.}}$ was larger than $Error_{PERP}^{K_{a,r}^{KAP,Hybrid}}$ and decreased sharply at a field size below 100 cm^2^. In the clinical simulation, although both $Error_{PERP}^{K_{a,r}^{KAP,Meas.}}$ and $Error_{skin}^{K_{a,r}^{KAP,Meas.}}$ were within the specified tolerance for the 30 × 30 cm^2^ and 15 × 15 cm^2^ FOVs, they far exceeded the specified tolerance at the 3.8 × 3.8 cm^2^ FOV. Therefore, $K_{a,r}^{KAP,Meas.}$ must be employed with X‐ray field size corrections when used as a surrogate for $K_{a,r}$ and $K_{a,skin}$. Furthermore, the aPERP for the whole cerebral angiography proposed in this study, located 11 cm from the isocenter toward the X‐ray focus, is useful for minimizing discrepancies between $K_{a,r}$ (or $K_{a,aPERP}$) and $K_{a,skin}$ during the whole cerebral angiography. In conclusion, the selection between these two metrics should be strategically guided by the specific clinical workflow: $K_{a,r}^{KAP,Hybrid}$ remains an acceptable surrogate for standard, wide‐field diagnostic cerebral angiography, whereas $K_{a,r}^{KAP,Meas.}$ with our proposed X‐ray field size and distance corrections provides vastly superior accuracy for highly collimated interventional neuroradiological procedures.

## AUTHOR COTRIBUTIONS

Atsushi Fukuda, Nao Ichikawa, and Takuma Hayashi contributed to the conception and design of the study, analysis and interpretation of data, collection, and assembly of data, drafting of the article, and final approval of the article. Pei‐Jan Paul Lin, Ayaka Hirosawa, Kosuke Matsubara contributed to the conception and design of the study, drafting of the article, and final approval of the article.

## FUNDING INFORMATION

Dr Fukuda has received research support from Toreck Corporation.

## CONFLICT OF INTEREST STATEMENT

Dr. Fukuda was provided with a research fund from Toreck Corporation to improve the accuracy of the KAP meter.

## ETHICS STATEMENT

This study did not involve human participants or animals. Therefore, institutional review board approval was not required.

## Data Availability

The data that support the findings of this study are available from the corresponding author upon reasonable request.
